# Coupled-Cluster Density-Based Many-Body Expansion

**DOI:** 10.1021/acs.jpca.3c04591

**Published:** 2023-10-23

**Authors:** Kevin Focke, Christoph R. Jacob

**Affiliations:** Institute of Physical and Theoretical Chemistry, Technische Universität Braunschweig, Gaußstraße 17, 38106 Braunschweig, Germany

## Abstract

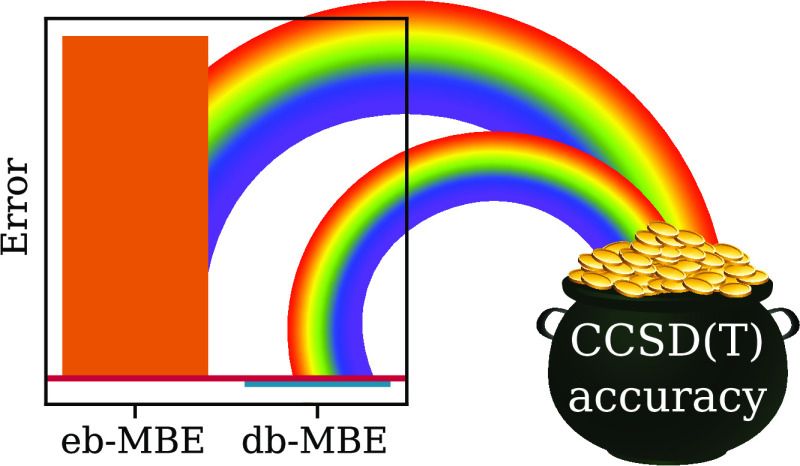

While CCSD(T) is often considered the “gold standard” of computational chemistry, the scaling of its computational cost as N^7^ limits its applicability for large and complex molecular systems. In this work, we apply the density-based many-body expansion [Int. J. Quantum Chem.2020, 120, e26228] in combination with CCSD(T). The accuracy of this approach is assessed for neutral, protonated, and deprotonated water hexamers, as well as (H_2_O)_16_ and (H_2_O)_17_ clusters. For the neutral water clusters, we find that already with a density-based two-body expansion, we are able to approximate the supermolecular CCSD(T) energies within chemical accuracy (4 kJ/mol). This surpasses the accuracy that is achieved with a conventional, energy-based three-body expansion. We show that this accuracy can be maintained even when approximating the electron densities using Hartree–Fock instead of using coupled-cluster densities. The density-based many-body expansion thus offers a simple, resource-efficient, and highly parallelizable approach that makes CCSD(T)-quality calculations feasible where they would otherwise be prohibitively expensive.

## Introduction

Coupled cluster methods^1^ allow for the quantum-chemical treatment of molecular systems with high accuracy. In particular, the CCSD(T) model^2^ in combination with sufficiently large basis sets is often considered the “gold standard” of computational chemistry, as it mostly provides outstanding accuracy at a relatively moderate computational cost.^3−5^ Nonetheless, its steep computational scaling (with the seventh power of the number of basis functions) considerably restricts its applicability for large and complex chemical systems.

The use of linear-scaling techniques,^6−8^ in particular the use of domain-based local pair natural orbitals (DLPNOs),^9,10^ provides one possibility for enabling the application of coupled-cluster methods such as CCSD(T) to larger systems (for examples, see, e.g., refs (11,12)). An alternative route is provided by fragmentation methods,^13−20^ which partition a large molecular system into smaller fragments that can each be treated individually.

Molecular clusters are particularly suited for the application of such fragmentation methods, as their constituting molecules define a natural partitioning into molecular fragments. Here, the many-body expansion (MBE)^21−23^ provides a straightforward and systematically improvable fragmentation method. It approximates the total energy of a molecular cluster in terms of the energies of monomers, dimers, trimers, and so on. These can each be calculated with accurate quantum-chemical methods such as CCSD(T).^24,25^ If this expansion can be truncated at sufficiently low order – preferably already at the two-body level (i.e., requiring only calculations for monomers and dimers) – it provides an attractive scheme for overcoming the steep scaling of the computational effort with coupled-cluster methods (for impressive examples, see refs (26,27)). Moreover, the individual fragment calculations can be executed in a massively parallel fashion (see, e.g., ref (28)).

However, the convergence of the MBE is often slow and the inclusion of three-body and possibly even four-body contributions can be necessary to achieve sufficient accuracy.^29−31^ Therefore, several strategies exist for accelerating the convergence of the MBE, such as the application of suitable embedding schemes in the fragment calculations,^32−34^ the use of multi-level schemes to approximate higher-order contributions,^35−38^ and the use of overlapping fragments.^39−41^

Motivated by the observation that an MBE of the electron density seems to converge faster with the expansion order than a conventional energy-based MBE (eb-MBE), our group devised the density-based MBE (db-MBE).^42^ It is inspired by subsystem density-functional theory (DFT)^43^ and applies the total energy functional of DFT to the many-body expanded electron density. This way, a density-dependent correction to the conventional eb-MBE can be derived that accounts for higher-order interactions between the fragments by means of orbital-free DFT.

Previously, we applied this db-MBE in combination with DFT calculations for the fragments and assessed its accuracy for water clusters^44^ as well as ion–water clusters.^45^ We showed that a two-body db-MBE already achieves an excellent accuracy for these test cases, which generally rivals the accuracy of a three-body eb-MBE.

However, the db-MBE is not restricted to DFT calculations. Both the energies used for the underlying eb-MBE and the electron densities used in the db-MBE can be obtained from any quantum-chemical method. Here, we combine the db-MBE with quantum-chemical calculations using CCSD(T). We present a flexible implementation that is able to import the relevant electron densities from various quantum-chemical program packages, and we assess the accuracy of the coupled-cluster db-MBE for neutral as well as protonated and deprotonated water clusters.

## Computational Methodology

### Many-Body Expansion: Energy-Based and Density-Based

In the conventional, energy-based many-body expansion (MBE),^22,23^ the total energy of a molecular cluster is expanded in terms of the energies of its *n*-mer fragments as,
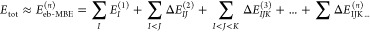
1where *E*_*I*_^(1)^ is the energy of the *I*-th fragment, Δ*E*_*IJ*_^(2)^ = *E*_*IJ*_^(2)^ – *E*_*I*_^(1)^ – *E*_*J*_^(1)^ is the interaction energy for the dimer of fragments *I* and *J*, and Δ*E*_*IJK*_^(3)^ is the trimer interaction energy. For explicit expressions for the *k*-mer interaction energies Δ*E*_*IJK*_^(*k*)^..., see, e.g., ref (46).

We refer to the truncation of eq 1 at order *n* as eb-MBE(*n*). For a given truncation of the MBE, the total number of calculations *N* that have to be carried out when applied to a system consisting of *N* fragments amounts to . Thus, truncating the MBE as early as possible extends the system size that can be treated for a given amount of computational resources. Here, we aim at achieving chemical accuracy (i.e., an error in the total energy below 4 kJ/mol) already at the level of a two-body expansion.

As only the total energies of the quantum-chemical calculations for the monomers, dimers, trimers etc. enter in the eb-MBE [see eq 1], it is straightforward to apply in combination with any quantum-chemical method. While for CCSD(T), the computational effort scales as *N*^7^, for a truncated *n*-body expansion the computational effort will only scale as *N*^*n*^. Thus, if the eb-MBE can be truncated already at the two-body level (resulting in an overall scaling as *N*^2^) without a substantial loss in accuracy, huge computational savings are possible. In addition, for the MBE, the underlying single-point calculations are massively parallelizable, as all fragment calculations are independent from each other.

Besides the total energy, also the total electron density can be approximated as
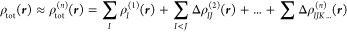
2where the monomer, dimer, trimer, etc. contributions are defined in analogy to the corresponding energy terms.

To improve upon the eb-MBE, we recently proposed a density-based many-body expansion (db-MBE),^44,45^ which is motivated by the observation that the MBE of the electron density [eq 2] seems to converge faster than the MBE of the total energy [eq 1].^42^ The db-MBE represents a generalization of subsystem DFT^43^ and can be formulated as an ONIOM-style multilevel approach,^47,48^ in which the eb-MBE serves as the high level, while orbital-free DFT is used as the low level, i.e.,
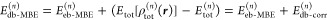
3Here, *E*_tot_[ρ]= *T*_*s*_[ρ]+ *V*_nuc_[ρ]+ *J*[ρ]+ *E*_xc_[ρ]+ *E*_NN_ is the total energy functional as defined in the framework of Kohn–Sham (KS) DFT, with the noninteracting kinetic energy *T*_*s*_[ρ], the electron–nuclei attraction energy *V*_nuc_[ρ], the Coulomb repulsion energy *J*[ρ], and the exchange–correlation energy *E*_xc_[ρ]. The MBE of the KS-DFT total energy functional *E*_tot_^(*n*)^ is defined as
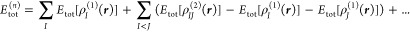
4i.e., the total energy functional is evaluated for the individual monomer, dimer, trimer, etc. electron densities.

The density-based energy correction *E*_db-corr_^(*n*)^ appearing in eq 3 is given by
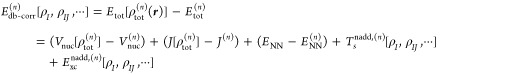
5with the *n*-body nonadditive kinetic and exchange-correlation energy functionals defined as
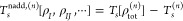
6
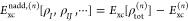
7Here, *V*_nuc_^(*n*)^, *J*^(*n*)^, *E*_NN_^(*n*)^, *T*_*s*_^(*n*)^, and *E*_xc_^(*n*)^ are the MBEs of the individual contributions to the KS-DFT total energy functional, which are defined in analogy to eq 4. Note that a correction due to the nuclear repulsion energy only appears at first order because *E*_NN_^(*n*)^ = *E*_NN_ for *n* ≤ 2. In our implementation, the contributions of the electron–nuclei attraction and of the Coulomb repulsion are evaluated recursively order by order.^42^

As long as the contributions of the nonadditive kinetic and exchange–correlation energies [eqs 6 and 7] are approximated using (semi)local density functionals, all terms in eq 5 can be evaluated using the coordinates of the nuclei and the electron densities of the monomers, dimers, trimers, etc. only. Therefore, while in our previous work, we applied the db-MBE only in combination with DFT calculations,^42,44,45^ it can be extended to any quantum-chemical method that provides an electron density, including wave function-based methods such as coupled cluster theory.

We note that while it is possible to accelerate the convergence of both the eb-MBE and the db-MBE by using a suitable embedding potential in the quantum-chemical calculation of the monomers, dimers, trimers, etc. (see ref (42) for a discussion), no embedding is considered in the present work. Instead, all fragment calculations are executed for the isolated molecules.

### Evaluation of the Density-Based Correction

The calculation of the density-based correction *E*_db-corr_^(*n*)^ [eq 5] requires the evaluation of the density functionals
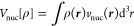
8and
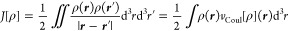
9with , as well as
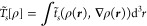
10and
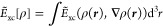
11where the tilde denotes the use of a semilocal density functional approximation. Given the electron density ρ(*r*_*k*_) at suitable grid points *r*_*k*_, the electron–nuclei interaction energy *V*_nuc_[ρ] can be obtained by numerical integration of eq 8. The numerical evaluation of *T̃*_*s*_[ρ] and *Ẽ*_xc_[ρ] [eqs 10 and 11] additionally requires the density gradient ***∇***ρ(***r***_*k*_) at the grid points. Finally, for an efficient numerical integration of eq 9, the Coulomb potential *v*_Coul_[ρ](***r***_*k*_) at each grid point must be calculated analytically.

For single-determinant wave functions, most importantly for Hartree–Fock (HF) and DFT, the electron density is (for a closed-shell molecule with doubly occupied orbitals) given by,
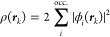
12where the molecular orbitals ϕ_*i*_ are commonly expanded in basis functions. This makes the electron density as well as its gradient at the grid points straightforward to evaluate. With analytical integrals of the Coulomb operator over the basis functions available, the Coulomb potential at the grid points can also be calculated analytically.

With coupled-cluster methods, such as CCSD(T), the calculation of the electron density is not as easy because the coupled-cluster wave function is not variational and the expansion of the exponential operator *e*^*T̂*^ in the expectation value only terminates when all possible excitation levels are exhausted.^49^ Thus, a direct calculation of the electron density requires using a linear-response formulation, which is computationally expensive.^50^

An alternative is provided by the orbital-optimized coupled-cluster doubles (OO–CCD) approach,^51−53^ in which the single-excitation operator *T̂*_1_ is replaced by an orbital rotation operator κ̂, leading to the introduction of orbital relaxation effects into the theory. The OO–CCD energy is then obtained from the stationary condition with respect to both the doubles amplitudes *t*_*ab*_^*ij*^ and the orbital rotation parameters κ_*ai*_, i.e.,
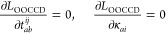
13where *L*_OOCCD_ is the OO–CCD Lagrangian.^53^ This leads to the orbital-optimized coupled-cluster doubles equations, which include both the doubles amplitude equations and the orbital rotation equations. Solving these equations self-consistently provides the OO–CCD energy, as well as a reference determinant with optimized orbitals. This optimized reference determinant can then be used to obtain an electron density according to eq 12 as well as the corresponding Coulomb potential.

The OO–CCD orbitals are very similar to the (nonoptimized) Brueckner orbitals,^54−56^ and it has been shown previously that the corresponding electron density and associated molecular properties are generally more accurate than those obtained from the Hartree–Fock determinant.^55,57−59^

### Implementation

Previously, the db-MBE was implemented in our scripting framework PyAdf(60) in combination with DFT calculations using the Amsterdam Modeling Suite (Ams).^61,62^ For details, we refer to the description in ref (42). In this case, we made use of Ams’s own tools for exporting electron densities and Coulomb potentials on an integration grid, which had previously been implemented to allow for the export of embedding potentials.^63,64^

While PyAdf is interfaced to numerous quantum-chemical program packages, this interface was specific to Ams. Therefore, we decided to implement a more general, high-level interface that abstracts the handling of the electron densities and Coulomb potentials on a grid from the calculation of these densities. This general interface then allows for an evaluation of the density-based correction [eq 5] that is agnostic to the source of the electron densities and Coulomb potentials.

To access densities from quantum-chemical program packages that use Gaussian-type orbitals (GTOs), we make use of the Molden file format.^65^ While modern file formats^66,67^ offer more versatile functionality, Molden files are exported by all common quantum-chemical program packages. We note that in the case of Orca,^68,69^ the generated Molden files are not consistent with the commonly used format for GTOs with high angular momentum. Therefore, Orca’s Molden files are modified following ref (70).

Molden files generated by quantum-chemical program packages are then read with the help of an interface to PyScf,^71,72^ which also calculates the densities and Coulomb potentials at the required grid points. These are then fed into the general interface for evaluating the density-based energy correction. To prevent redundant recalculations, we leveraged the HDF5 file format^73^ and the h5py package^74^ extensively in our code for storing densities and Coulomb potentials. We note that while in the present work, we only include calculations using Orca, our implementation is general and can be applied in combination with any quantum-chemical program package supported by PyAdf.

All required quantum-chemical calculations – both for the eb-MBE and possibly also for generating the electron densities and Coulomb potentials required for the db-MBE, are executed by PyAdf. While the generation of densities and potentials as well as the evaluation for the density-based correction require some computational overhead, they do not increase the scaling of the computational effort with the number of fragments. We further note that while a massive parallelization of the eb-MBE and db-MBE is in principle trivial and work toward enabling the parallel execution of the individual quantum-chemical calculations in PyAdf is currently in progress in our group, we did not make use of parallelism in the present work.

### Computational Details

The molecular structures of all considered structures have been taken from refs (75−78) and are also available in the Supporting Information of refs (44,45).

All calculations were performed with a development version of PyAdf,^60,79^ employing the Orca program package^68,69^ (Version 5.0.3) for all quantum-chemical calculations. For the calculation of electron densities and Coulomb potentials, PyAdf is interfaced to PyScf(80) as described above.

All quantum-chemical calculations were performed using Dunning’s aug-cc-pVTZ basis set.^81^ The DFT calculations have been performed with the Perdew–Burke–Ernzerhof (PBE) exchange–correlation functional.^82^ In all quantum-chemical calculations, Orca’s “very tight” self-consistent field convergence criteria were chosen. The CCSD(T) and OO–CCD calculations otherwise used Orca’s default settings. In particular, the oxygen 1*s* core orbitals are frozen in the coupled-cluster treatment.

For the evaluation of the density-based correction [eq 5], we employ a supermolecular integration grid that is exported from Ams,^62^ using its default (basic) Becke grid.^83^ For the nonadditive exchange–correlation energy, the PBE functional^82^ is employed, whereas for the nonadditive kinetic energy, the PW91k approximation^84^ is used throughout.

## Results and Discussion

### (H_2_O)_6_

**Figure 1:**
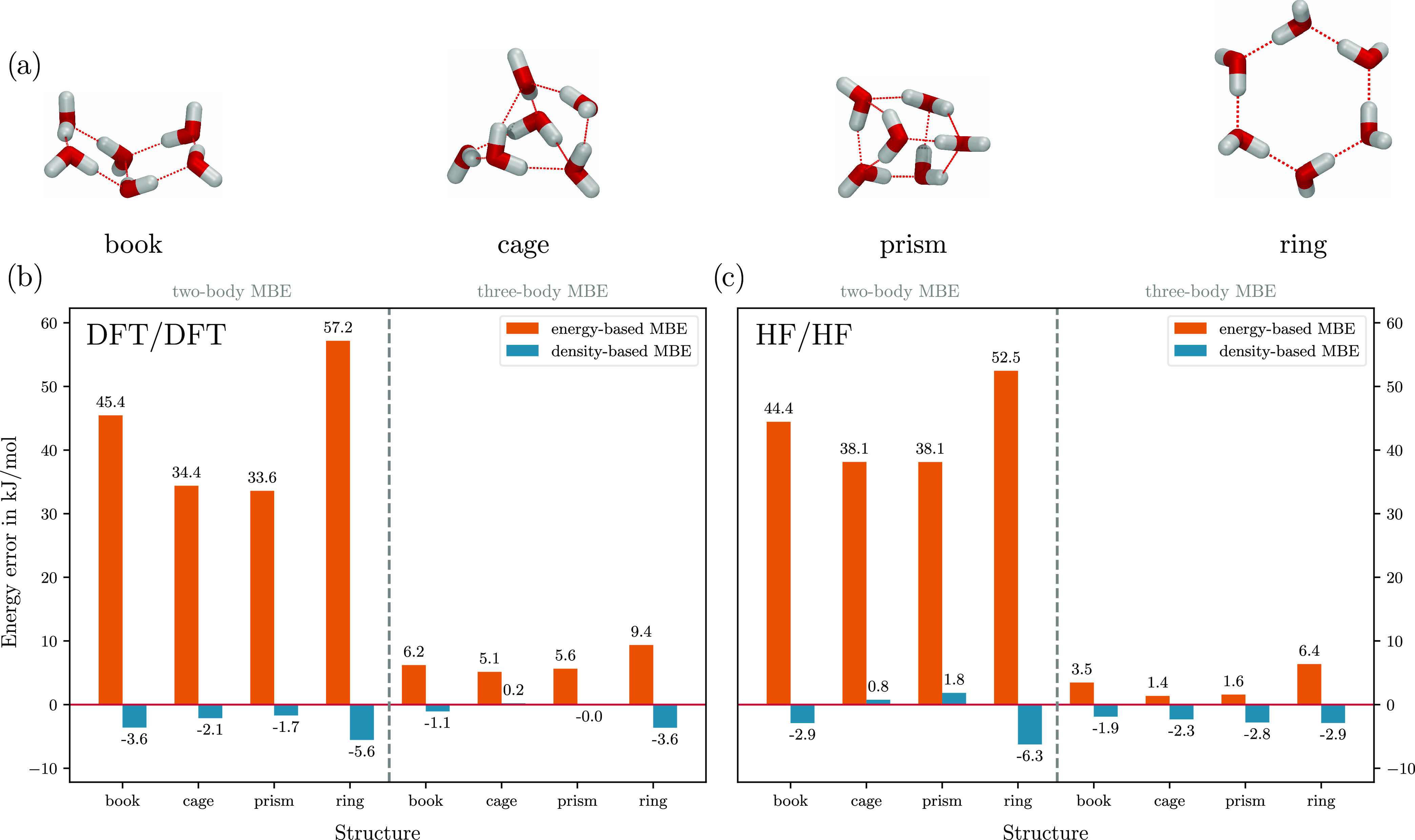
(a) Molecular structures of four low-lying isomers of (H_2_O)_6_. (b, c) Errors in their total energies obtained with the two-body and three-body eb-MBE as well as db-MBE compared to the full, supermolecular calculations using (b) DFT (with PBE xc functional) and (c) HF and the aug-cc-pVTZ basis set. Here, both the eb-MBE and the density-based correction are evaluated using the same method.

As a first test case, we consider the four low-lying isomers of the water hexamer, (H_2_O)_6_. The molecular structures have been taken from ref (75) and are shown in Figure 1a. This test case has been used previously by our group to assess the accuracy of the db-MBE in combination with DFT and our previous implementation using PyAdf and Ams.^44^

Figure 1b compares the errors of the two-body and three-body eb-MBE as well as db-MBE for the four (H_2_O)_6_ isomers. While with the eb-MBE(2), errors between 33.6 and 57.2 kJ/mol are found, the db-MBE(2) reduces these errors to below 5.6 kJ/mol. With the eb-MBE(3), the errors amount to only 5.1–9.4 kJ/mol, but the db-MBE(3) reduces the error further to below 3.6 kJ/mol. For the book, cage, and prism isomers, the errors even remain below 1.1 kJ/mol. Note that for the ring isomer, higher-order effects are particularly large.^85^

These results obtained with Orca and our new PyAdf implementation are very similar to those reported previously in ref (44) with Ams and PyAdf, even though a different basis set has been used. As our new implementation in PyADF is agnostic to the origin of the energies and of the Molden files it processes to obtain the electron densities and Coulomb potentials, we can infer that our implementation should be working correctly also in combination with other quantum-chemical methods and program packages.

The same scheme was seamlessly applied to the same water hexamers employing the Hartree–Fock (HF) method. In this case, HF energies are used in the eb-MBE as well as the reference [eq 1], and HF densities are used for evaluating the density-based correction [eq 5]. The errors are visualized in Figure 1c.

Overall, the errors with HF are very similar to those observed in the case of DFT, in particular for the two-body expansion. With the db-MBE(2), the errors are between 0.8 and 6.3 kJ/mol. With the eb-MBE(3), the errors are slightly smaller with HF than with DFT, while with the db-MBE(3), they are slightly larger. This might indicate a slight mismatch between the density-based correction (which makes use of an approximate nonadditive exchange–correlation functional) and the supermolecular HF calculation, which does not include correlation effects.

For DFT and HF, our results confirm that the db-MBE(2) can drastically reduce the error compared to the eb-MBE(2) and can achieve results of similar accuracy as the eb-MBE(3). With the exception of the ring isomer, the errors of the db-MBE(2) are below the threshold of chemical accuracy. We note that the accuracy of both the eb-MBE and the db-MBE could be further improved with an adequate implementation of embedding schemes, such as point charges and frozen density embedding.^42,44^

Next, we extended our tests to CCSD(T) calculations for (H_2_O)_6_. For the eb-MBE [eq 1], we used the CCSD(T) total energies, whereas for evaluating the density-based correction [eq 5], we used the densities from separate OO–CCD calculations. These densities should be reasonably close to the true CCSD(T) densities, which are not available.

**Figure 2:**
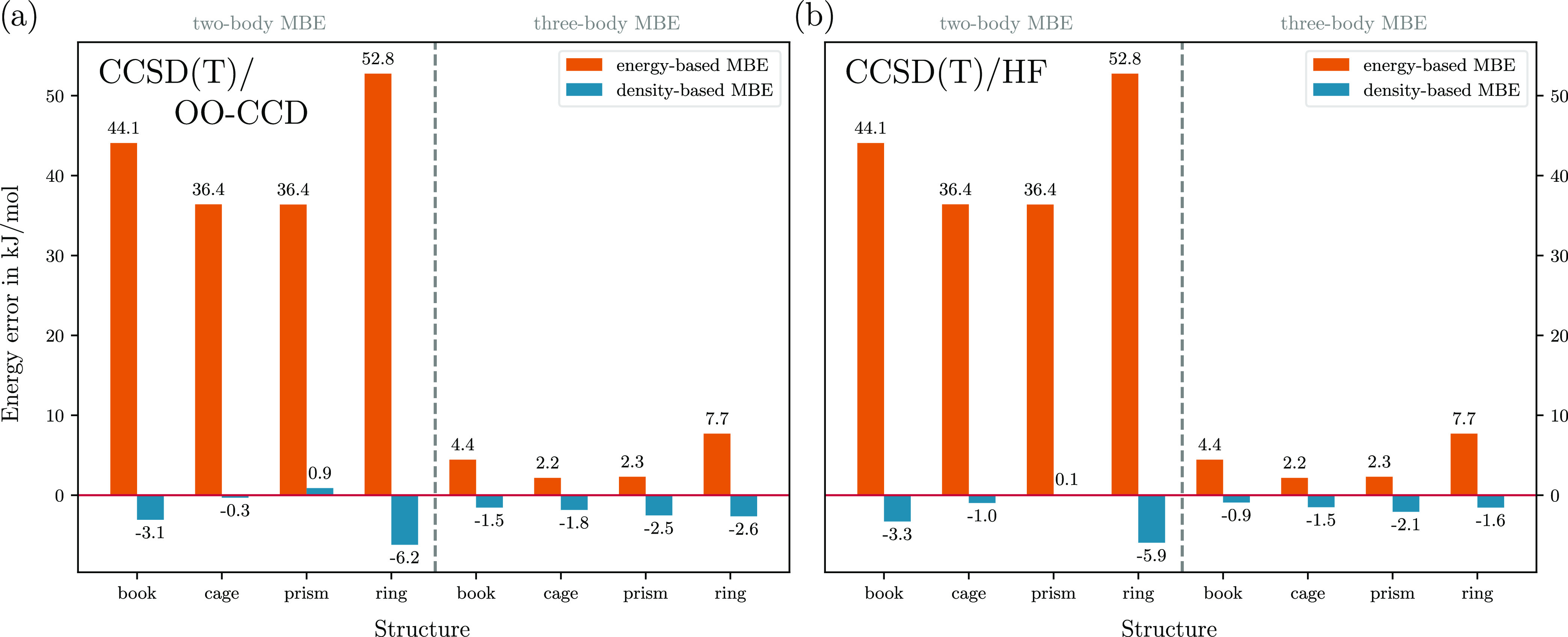
Errors in the total energies of the four low-lying isomers of (H_2_O)_6_ obtained with the two- and three-body eb-MBE as well as db-MBE compared to the full, supermolecular CCSD(T)/aug-cc-pVTZ calculations. The eb-MBE is performed using CCSD(T)/aug-cc-pVTZ for the fragment calculations, whereas the density-based correction of the db-MBE is evaluated using (a) OO–CCD densities and (b) HF densities.

The resulting errors are shown in Figure 2a. The accuracy is very similar to the one observed with DFT and with HF. While the eb-MBE(2) produces errors of up to 52.8 kJ/mol, the db-MBE(2) is able to reduce the error to below 6.2 kJ/mol for all four isomers. The eb-MBE(3) achieves similar accuracy as the db-MBE(2), with errors of up to 7.7 kJ/mol. The db-MBE(3) further reduces the error to well below chemical accuracy (smaller than 2.6 kJ/mol for all isomers). Thus, the high accuracy of the db-MBE(2) and db-MBE(3) is preserved when using CCSD(T) instead of DFT.

However, the OO–CCD(T) calculations proved costly, and there were significant problems with convergence, especially with the larger fragments at higher orders. Calculations took between 2 and 10 times longer than the corresponding CCSD(T) calculations, depending on the convergence and the size of the fragment. Therefore, we also explored approaches using cheaper methods to obtain an electron density.

As CCSD(T) calculations take their reference determinant from an HF calculation, there is no additional effort involved in simply taking the density from there. Thus, we combined the eb-MBE obtained from the CCSD(T) energies with the density-based correction evaluated using the HF electron densities.

Figure 2b shows the errors of the db-MBE(2) and the db-MBE(3) obtained in this case. The results with OO–CCD densities and with HF densities are remarkably close, with differences below 1 kJ/mol. In fact, the errors are slightly reduced when using the HF densities, which is most likely due to error cancelation. In conclusion, HF densities seem to be a reasonable approximation of the CCSD(T) densities in the context of the db-MBE.

### (H_3_O)^+^(H_2_O)_5_ and (OH)^−^(H_2_O)_5_

**Figure 3:**
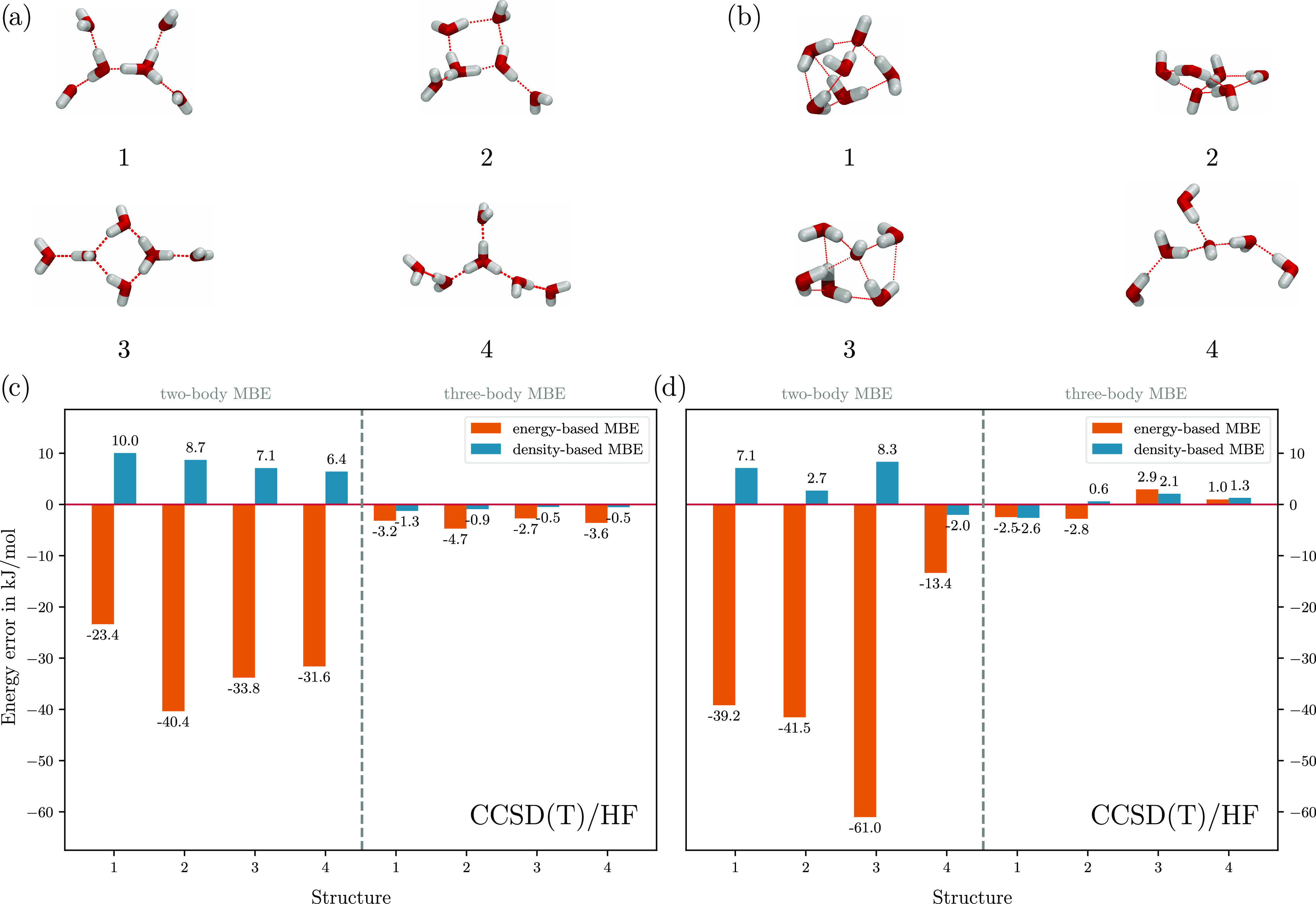
(a, b) Molecular structures of four low-lying isomers of (a) (H_3_O)^+^(H_2_O)_5_ and (b) (OH)^−^(H_2_O)_5_. (c, d) Errors in the total energies of (c) the (H_3_O)^+^(H_2_O)_5_ isomers and (d) the (OH)^−^(H_2_O)_5_ isomers obtained with the two-body and three-body eb-MBE (using CCSD(T)/aug-cc-pVTZ) as well as db-MBE (using HF densities) compared to the full, supermolecular CCSD(T)/aug-cc-pVTZ calculations.

At this stage, we turn to a more complex test case and consider protonated and deprotonated water hexamers (H_3_O)^+^(H_2_O)_5_ and (OH)^−^(H_2_O)_5_. In each case, we investigated four low-energy isomers (see Figure 3a,b) with structures taken from refs (77) and (76), respectively. These ion–water clusters are more challenging for the MBEs, since larger higher-order contributions can be expected due to the polarizability of the water molecules. Previously,^45^ we investigated the accuracy of the relative energies of these isomers of (H_3_O)^+^(H_2_O)_5_ and (OH)^−^(H_2_O)_5_ with the eb-MBE and the db-MBE in combination with DFT. As in this earlier work, each water molecule as well as (H_3_O)^+^ and (OH)^−^ are treated as separate fragments.

Here, we apply CCSD(T) for the eb-MBE and combine it with a density-based correction evaluated with HF densities for the db-MBE. The errors of the two-body and three-body eb-MBE and db-MBE for the protonated and deprotonated water hexamers are shown in Figure 3c,d. The eb-MBE(2) shows errors of up to 40.4 and 61.0 kJ/mol for (H_3_O)^+^(H_2_O)_5_ and (OH)^−^(H_2_O)_5_, respectively. These errors are comparable in magnitude to those for the neutral water hexamers (see Figure 2b), but the errors scatter more widely; i.e., the errors in the relative energies will be substantially larger.

With the db-MBE(2), the errors are reduced to a maximum of 10.0 and 8.3 kJ/mol for (H_3_O)^+^(H_2_O)_5_ and (OH)^−^(H_2_O)_5_, respectively. This, roughly twice as large as for the neutral hexamers, demonstrates the increased complexity of the charged clusters and the larger importance of higher-order contributions. As shown in ref (45). for ion–water clusters, the db-MBE greatly benefits from the use of embedding schemes in the fragment calculations, which improves the convergence of the electron densities and substantially lowers the error of the db-MBE(2).

Despite the observed uptick in error, the db-MBE(2) still offers a great improvement over the conventional, energy-based approach. With a three-body expansion, both the eb-MBE(3) and the db-MBE(3) approach chemical accuracy. For the protonated water hexamers, the db-MBE(3) further reduces the error to below 1.3 kJ/mol, while it offers little improvement for the deprotonated water hexamers. This indicates a minor discrepancy between the density-based correction [eq 5], which is calculated using approximate density functionals and HF densities, and the CCSD(T) reference to which it is compared. While the energy of the eb-MBE approaches the exact CCSD(T) value, the HF electron density used in the db-MBE scheme approaches neither an exact CC density nor the DFT density for which the approximate functionals are optimized. Despite this mismatch, the energy error formally approaches zero, as the db-MBE correction includes only the interaction and will ultimately approach zero at a sufficiently high order of the MBE.

### (H_2_O)_16_ and (H_2_O)_17_

**Figure 4:**
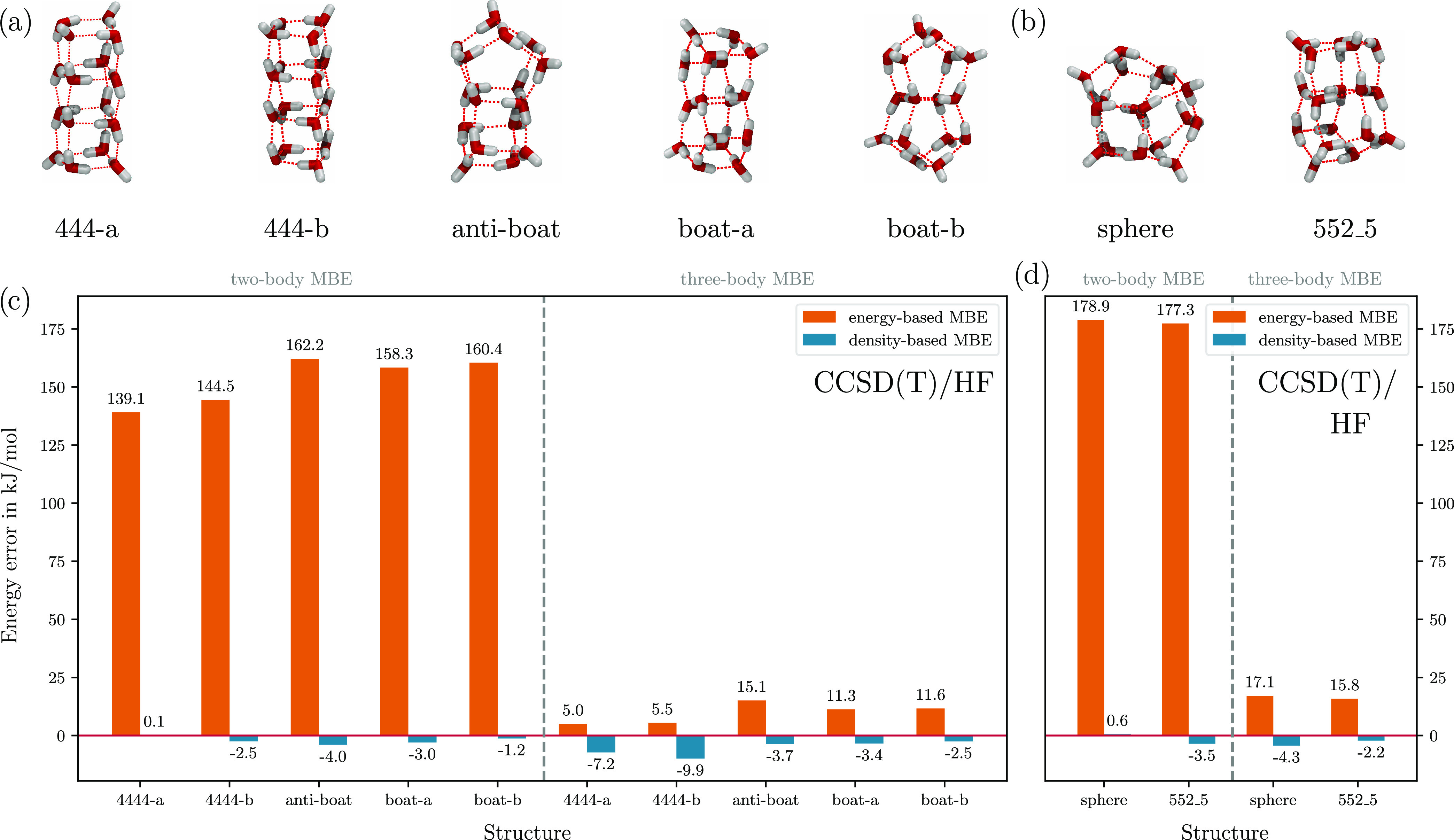
(a, b) Molecular structures of (a) five low-lying isomers of (H_2_O)_16_ and (b) two low-lying isomers of (H_2_O)_17_. (c, d) Errors in the total energies of (c) the (H_2_O)_16_ isomers and (d) the (H_2_O)_17_ isomers obtained with the two-body and three-body eb-MBE (using CCSD(T)/aug-cc-pVTZ) as well as db-MBE (using HF densities) compared to the full, supermolecular CCSD(T)/aug-cc-pVTZ calculations.

To conclude our series of tests, we expanded the scope of our investigation to encompass larger systems: specifically, five isomers of (H_2_O)_16_ and two isomers of (H_2_O)_17_. The molecular structures (see Figure 4a,b) have been taken from ref (78). For previous results using the eb-MBE and db-MBE based on DFT calculations, we refer to ref (44). Here, we again employ the eb-MBE based on CCSD(T) energies in combination with a density-based correction evaluated using HF densities.

The errors of the eb-MBE and the db-MBE compared to the supermolecular CCSD(T)/aug-cc-pVTZ reference energies published in ref (78) are plotted in Figure 4c,d. For the four considered isomers of (H_2_O)_16_, the eb-MBE(2) shows errors between 139.1 and 162.2 kJ/mol. For the two considered isomers of (H_2_O)_17_, we find errors of 177.3 and 178.9 kJ/mol. With the db-MBE(2), the errors are in all cases reduced below 4 kJ/mol, i.e., within the threshold of chemical accuracy. In all cases, this is substantially more accurate than the eb-MBE(3), which exhibits errors of up to 15.1 and 17.1 kJ/mol for (H_2_O)_16_ and (H_2_O)_17_, respectively.

While for the eb-MBE(2), the error for these larger water clusters increased substantially compared to that for the water hexamers, the db-MBE(2) appears to maintain similar accuracy independent of the cluster size. Note, however, that in a previous comparison for water clusters of increasing size utilizing DFT,^44^ we found that the error of the db-MBE(2) still increases with cluster size, albeit with a significantly reduced error per fragment compared to the eb-MBE(2). Therefore, the excellent agreement observed here might partly be due to error cancelation. This is also indicated by the observation that the errors slightly increase when going from the db-MBE(2) to the db-MBE(3). Again, this might be related to inconsistencies between the approximate density functionals and HF densities in the evaluation of the density-based correction.

Nevertheless, the db-MBE clearly offers a substantial improvement over the conventional eb-MBE in all cases, and already the two-body db-MBE(2) is able to approach the threshold of chemical accuracy, even for larger water clusters.

## Conclusions

In the present work, we assessed the accuracy of the db-MBE in combination with CCSD(T) for neutral, protonated, and deprotonated water clusters. To arrive at an efficient scheme and to reduce the computational overhead of the db-MBE compared to the eb-MBE, we replace the use of coupled-cluster densities by HF densities and show that this is possible without noticeable loss in accuracy. Nevertheless, a description of the density that includes electron correlation effects might still prove useful for systems in which these play a more prominent role, and other efficient ways of generating coupled-cluster densities, such as using Brueckner orbitals, remain to be explored.

For the neutral water clusters, we have consistently shown that the db-MBE(2) outperforms the conventional eb-MBE(3), i.e., the convergence of the MBE can be accelerated by one order. The db-MBE(2) is generally able to reduce the error compared to a full, supermolecular CCSD(T) treatment below the threshold of chemical accuracy (4 kJ/mol). Therefore, we are confident that the db-MBE will enable accurate coupled-cluster calculations for large and complex molecular systems and plan to explore these possibilities for biomolecular systems^20^ in our future work.

When applied to small charged systems, the db-MBE(2) displayed slightly less accurate results compared to the eb-MBE(3). Here, higher-order contributions become more important and lead to an increased error. However, we expect that such issues can be mitigated by combining our db-MBE with suitable embedding schemes. This was previously shown for the db-MBE in combination with DFT,^45^ but the consistent inclusion of an embedding potential in wave function-based quantum-chemical calculations for use in the eb-MBE and db-MBE is more challenging to implement (see the discussion in ref (42)).

## Data Availability

The data underlying this study are available within this article and its Supporting Information.

## Supporting Information Available

Total interaction energies obtained with the eb-MBE and db-MBE as well as with supermolecular calculations for all considered water clusters (PDF)
