# Supplementary material for: Coupled-Cluster Density-Based Many-Body Expansion
Source: J Phys Chem A. 2023 Oct 23;127(43):9139–48. doi: 10.1021/acs.jpca.3c04591 (PMC10626589; doi:10.1021/acs.jpca.3c04591)
Supplement: Supplementary file 1 — jp3c04591_si_001.pdf [file jp3c04591_si_001.pdf]

Coupled-Cluster Density-Based Many-Body Expansion

Kevin Focke, Christoph R. Jacob*[,1]

Technische Universität Braunschweig,

Institute of Physical and Theoretical Chemistry,

Gaußstraße 17, 38106 Braunschweig, Germany

Supporting Information

[1]E-Mail: c.jacob@tu-braunschweig.de

S1 Interaction energies at different orders

This Supporting Information contains interaction energies for the calculations presented in the results section of the main article. The total interaction of a system $E_{\text{int,tot}}^{\text{super}}$ describes the interaction between the subsystems. It is defined as

$$E_{\text{int,tot}}^{\text{super}} = E_{\text{tot}}^{\text{super}} - \sum_I E_I^{(1)} = E_{\text{tot}}^{\text{super}} - E_{\text{tot}}^{\text{eb-MBE(1)}}. \tag{S1}$$

Herein, $E_I^{(1)}$ is the energy of an isolated subsystem, i.e. a monomer, and $E_{\text{tot}}^{\text{super}}$ is the total energy of the full system in a supermolecular calculation at the considered level of theory. For $(H_2O)_{16}$ and $(H_2O)_{17}$, the latter values have been taken from Ref. [1].

Within the energy-based MBE scheme discussed in the paper, the sum of isolated monomer energies $\sum_I E_I^{(1)}$ is equivalent to the one-body expansion $E_{\text{tot}}^{\text{eb-MBE(1)}}$. The other interaction energies at the different orders n of the MBE are approximations of this total interaction energy and defined analogously as

$$E_{\text{int,tot}}^{\text{super}} \approx E_{\text{int,tot}}^{\text{eb-MBE(n)}} = E_{\text{tot}}^{\text{eb-MBE(n)}} - E_{\text{tot}}^{\text{eb-MBE(1)}} \tag{S2}$$

and

$$E_{\text{int,tot}}^{\text{super}} \approx E_{\text{int,tot}}^{\text{db-MBE(n)}} = E_{\text{tot}}^{\text{db-MBE(n)}} - E_{\text{tot}}^{\text{eb-MBE(1)}}. \tag{S3}$$

Errors, as presented in the main text, can be calculated as differences between the approximated total interaction energies and the exact value for $E_{\text{int,tot}}^{\text{super}}$. Results achieved with various methods as applied to the neutral water hexamers are presented in tables S1 to S4. Our other test systems are presented only with the preferred combination of CCSD(T) energies and HF densities. They can be found in tables S5 to S8.

Table S1: Total interaction energies in **kJ/mol** calculated for the neutral water hexamers $\mathbf{(H_2O)_6}$ from: the full calculation of the cluster ($E^{\text{super}}_{\text{int,tot}}$), the energy-based MBE of order n ($E^{\text{eb-MBE(n)}}_{\text{int,tot}}$) and the density-based MBE of order n ($E^{\text{db-MBE(n)}}_{\text{int,tot}}$). The calculations for the reference energies and the energy-based MBE energies are done with **DFT/PBE/aug-cc-pVTZ**. The density-based energies start from the energy-based MBE energies and use **DFT/PBE/aug-cc-pVTZ** densities and **PBE/PW91k** for the corrections.

		$E^{\text{eb-MBE(n)}}_{\text{int,tot}}$					$E^{\text{db-MBE(n)}}_{\text{int,tot}}$					
Cluster	$E^{\text{super}}_{\text{int,tot}}$	eb(2)	eb(3)	eb(4)	eb(5)	eb(6)	db(1)	db(2)	db(3)	db(4)	db(5)	db(6)
book	−196.83	−151.38	−190.62	−196.75	−196.80	−196.83	−96.48	−200.43	−197.89	−196.62	−196.58	−196.68
cage	−193.94	−159.55	−188.79	−194.32	−193.92	−193.94	−108.55	−196.07	−193.74	−194.25	−194.03	−194.13
prism	−193.34	−159.75	−187.70	−194.39	−193.25	−193.34	−113.35	−195.05	−193.38	−193.21	−193.37	−193.47
ring	−195.48	−138.32	−186.12	−194.68	−195.45	−195.48	−84.35	−201.04	−199.12	−195.76	−195.38	−195.52

Table S2: Total interaction energies in **kJ/mol** calculated for the neutral water hexamers $\mathbf{(H_2O)_6}$ from: the full calculation of the cluster ($E^{\text{super}}_{\text{int,tot}}$), the energy-based MBE of order n ($E^{\text{eb-MBE(n)}}_{\text{int,tot}}$) and the density-based MBE of order n ($E^{\text{db-MBE(n)}}_{\text{int,tot}}$). The calculations for the reference energies and the energy-based MBE energies are done with **HF/aug-cc-pVTZ**. The density-based energies start from the energy-based MBE energies and use **HF/aug-cc-pVTZ** densities and **PBE/PW91k** for the corrections.

		$E^{\text{eb-MBE(n)}}_{\text{int,tot}}$					$E^{\text{db-MBE(n)}}_{\text{int,tot}}$					
Cluster	$E^{\text{super}}_{\text{int,tot}}$	eb(2)	eb(3)	eb(4)	eb(5)	eb(6)	db(1)	db(2)	db(3)	db(4)	db(5)	db(6)
book	−139.95	−95.51	−136.48	−139.84	−139.93	−139.95	−147.82	−142.86	−141.84	−139.84	−139.80	−139.80
cage	−134.53	−96.40	−133.16	−134.48	−134.53	−134.53	−159.86	−133.77	−136.83	−134.60	−134.71	−134.72
prism	−134.65	−96.54	−133.08	−134.76	−134.65	−134.65	−163.76	−132.81	−137.46	−134.33	−134.79	−134.78
ring	−144.18	−91.73	−137.82	−143.66	−144.15	−144.18	−135.42	−150.43	−147.07	−144.53	−144.22	−144.22

Table S3: Total interaction energies in **kJ/mol** calculated for the neutral water hexamers $\mathbf{(H_2O)_6}$ from: the full calculation of the cluster ($E^{\text{super}}_{\text{int,tot}}$), the energy-based MBE of order n ($E^{\text{eb-MBE(n)}}_{\text{int,tot}}$) and the density-based MBE of order n ($E^{\text{db-MBE(n)}}_{\text{int,tot}}$). The calculations for the reference energies and the energy-based MBE energies are done with **CCSD(T)/aug-cc-pVTZ**. The density-based energies start from the energy-based MBE energies and use **OO-CCD/aug-cc-pVTZ** orbital-densities and **PBE/PW91k** for the corrections.

		$E^{\text{eb-MBE(n)}}_{\text{int,tot}}$					$E^{\text{db-MBE(n)}}_{\text{int,tot}}$		
Cluster	$E^{\text{super}}_{\text{int,tot}}$	eb(2)	eb(3)	eb(4)	eb(5)	eb(6)	db(1)	db(2)	db(3)
book	−202.45	−158.38	−198.00	−202.42	−202.41	−202.45	−130.57	−205.53	−203.99
cage	−205.11	−168.72	−202.94	−205.32	−205.10	−205.11	−142.97	−205.42	−206.95
prism	−206.50	−170.13	−204.19	−206.96	−206.43	−206.50	−146.94	−205.61	−209.02
ring	−196.83	−144.08	−189.14	−196.16	−196.78	−196.83	−117.75	−203.03	−199.48

Table S4: Total interaction energies in **kJ/mol** calculated for the neutral water hexamers $\mathbf{(H_2O)_6}$ from: the full calculation of the cluster ($E^{\text{super}}_{\text{int,tot}}$), the energy-based MBE of order n ($E^{\text{eb-MBE(n)}}_{\text{int,tot}}$) and the density-based MBE of order n ($E^{\text{db-MBE(n)}}_{\text{int,tot}}$). The calculations for the reference energies and the energy-based MBE energies are done with **CCSD(T)/aug-cc-pVTZ**. The density-based energies start from the energy-based MBE energies and use **HF/aug-cc-pVTZ** orbital-densities and **PBE/PW91k** for the corrections.

		$E^{\text{eb-MBE(n)}}_{\text{int,tot}}$					$E^{\text{db-MBE(n)}}_{\text{int,tot}}$					
Cluster	$E^{\text{super}}_{\text{int,tot}}$	eb(2)	eb(3)	eb(4)	eb(5)	eb(6)	db(1)	db(2)	db(3)	db(4)	db(5)	db(6)
book	−202.45	−158.38	−198.00	−202.42	−202.41	−202.45	−147.82	−205.73	−203.36	−202.42	−202.27	−202.30
cage	−205.11	−168.72	−202.94	−205.32	−205.10	−205.11	−159.86	−206.08	−206.61	−205.43	−205.27	−205.30
prism	−206.50	−170.13	−204.19	−206.96	−206.43	−206.50	−163.76	−206.39	−208.57	−206.54	−206.57	−206.63
ring	−196.83	−144.08	−189.14	−196.16	−196.78	−196.83	−135.42	−202.78	−198.39	−197.03	−196.85	−196.87

Table S5: Total interaction energies in **kJ/mol** calculated for the protonated water hexamers $\mathbf{(H_3O^+)\text{-}(H_2O)_5}$ from: the full calculation of the cluster ($E^{\text{super}}_{\text{int,tot}}$), the energy-based MBE of order n ($E^{\text{eb-MBE(n)}}_{\text{int,tot}}$) and the density-based MBE of order n ($E^{\text{db-MBE(n)}}_{\text{int,tot}}$). The calculations for the reference energies and the energy-based MBE energies are done with **CCSD(T)/aug-cc-pVTZ**. The density-based energies start from the energy-based MBE energies and use **HF/aug-cc-pVTZ** orbital-densities and **PBE/PW91k** for the corrections.

		$E^{\text{eb-MBE(n)}}_{\text{int,tot}}$					$E^{\text{db-MBE(n)}}_{\text{int,tot}}$					
Cluster	$E^{\text{super}}_{\text{int,tot}}$	eb(2)	eb(3)	eb(4)	eb(5)	eb(6)	db(1)	db(2)	db(3)	db(4)	db(5)	db(6)
1	−508.20	−531.57	−511.37	−507.92	−508.20	−508.20	−286.45	−498.16	−509.45	−508.25	−508.18	−508.19
2	−456.72	−497.08	−461.44	−456.21	−456.73	−456.72	−336.19	−448.03	−457.63	−456.92	−456.70	−456.72
3	−455.17	−488.99	−457.89	−455.03	−455.19	−455.17	−339.97	−448.07	−455.66	−455.55	−455.14	−455.17
4	−449.23	−480.84	−452.83	−448.99	−449.23	−449.23	−324.50	−442.82	−449.76	−449.36	−449.23	−449.23

Table S6: Total interaction energies in **kJ/mol** calculated for the deprotonated water hexamers $\mathbf{(OH^-)\text{-}(H_2O)_5}$ from: the full calculation of the cluster ($E^{\text{super}}_{\text{int,tot}}$), the energy-based MBE of order n ($E^{\text{eb-MBE(n)}}_{\text{int,tot}}$) and the density-based MBE of order n ($E^{\text{db-MBE(n)}}_{\text{int,tot}}$). The calculations for the reference energies and the energy-based MBE energies are done with **CCSD(T)/aug-cc-pVTZ**. The density-based energies start from the energy-based MBE energies and use **HF/aug-cc-pVTZ** orbital-densities and **PBE/PW91k** for the corrections.

		$E^{\text{eb-MBE(n)}}_{\text{int,tot}}$					$E^{\text{db-MBE(n)}}_{\text{int,tot}}$					
Cluster	$E^{\text{super}}_{\text{int,tot}}$	eb(2)	eb(3)	eb(4)	eb(5)	eb(6)	db(1)	db(2)	db(3)	db(4)	db(5)	db(6)
1	−449.52	−488.71	−452.00	−450.09	−449.40	−449.52	−308.03	−442.42	−452.13	−449.46	−449.94	−449.90
2	−442.99	−484.53	−445.78	−444.26	−442.71	−442.99	−294.08	−440.30	−442.38	−442.68	−442.92	−442.90
3	−442.86	−503.88	−439.92	−443.18	−442.77	−442.86	−333.92	−434.54	−440.78	−443.00	−442.92	−442.88
4	−419.79	−433.14	−418.80	−421.32	−419.60	−419.79	−252.78	−421.81	−418.50	−419.48	−419.80	−419.75

Table S7: Total interaction energies in **kJ/mol** calculated for the water 16mers **$(H_2O)_{16}$** from: the full calculation of the cluster ($E^{\text{super}}_{\text{int,tot}}$), the energy-based MBE of order n ($E^{\text{eb-MBE(n)}}_{\text{int,tot}}$) and the density-based MBE of order n ($E^{\text{db-MBE(n)}}_{\text{int,tot}}$). The calculations for the reference energies and the energy-based MBE energies are done with **CCSD(T)/aug-cc-pVTZ**. The density-based energies start from the energy-based MBE energies and use **HF/aug-cc-pVTZ** orbital-densities and **PBE/PW91k** for the corrections.

		$E^{\text{eb-MBE(n)}}_{\text{int,tot}}$		$E^{\text{db-MBE(n)}}_{\text{int,tot}}$		
Cluster	$E^{\text{super}}_{\text{int,tot}}$	eb(2)	eb(3)	db(1)	db(2)	db(3)
4444-a	−738.62	−599.55	−733.62	−522.15	−738.48	−745.86
4444-b	−737.35	−592.86	−731.88	−516.49	−739.84	−747.24
anti-boat	−740.19	−578.02	−725.10	−500.80	−744.19	−743.88
boat-a	−744.10	−585.77	−732.84	−514.06	−747.09	−747.51
boat-b	−742.89	−582.47	−731.25	−519.23	−744.12	−745.42

Table S8: Total interaction energies in **kJ/mol** calculated for the water 17mers **$(H_2O)_{17}$** from: the full calculation of the cluster ($E^{\text{super}}_{\text{int,tot}}$), the energy-based MBE of order n ($E^{\text{eb-MBE(n)}}_{\text{int,tot}}$) and the density-based MBE of order n ($E^{\text{db-MBE(n)}}_{\text{int,tot}}$). The calculations for the reference energies and the energy-based MBE energies are done with **CCSD(T)/aug-cc-pVTZ**. The density-based energies start from the energy-based MBE energies and use **HF/aug-cc-pVTZ** orbital-densities and **PBE/PW91k** for the corrections.

		$E^{\text{eb-MBE(n)}}_{\text{int,tot}}$		$E^{\text{db-MBE(n)}}_{\text{int,tot}}$		
Cluster	$E^{\text{super}}_{\text{int,tot}}$	eb(2)	eb(3)	db(1)	db(2)	db(3)
sphere	−793.32	−614.45	−776.23	−516.35	−792.77	−797.63
552-5	−791.27	−613.96	−775.46	−532.09	−794.79	−793.47

References

[1] S. Yoo, E. Aprà, X. C. Zeng, and S. S. Xantheas, High-Level Ab Initio Electronic Structure Calculations of Water Clusters $(H_2O)_{16}$ and $(H_2O)_{17}$: A New Global Minimum for $(H_2O)_{16}$, *J. Phys. Chem. Lett.*, 2010, **1**, 3122–3127.
